# Vitamin D and Chronic Rhinosinusitis with Nasal Polyps: A Narrative Review and Perspectives

**DOI:** 10.3390/jcm14072467

**Published:** 2025-04-04

**Authors:** Adrien Philippart, Philippe Eloy

**Affiliations:** 1 Department of Oto-Rhino-Laryngology & Head and Neck Surgery, Saint-Jean Clinics, Boulevard du Jardin Botanique 32, 1000 Brussels, Belgium; 2 Department of Oto-Rhino-Laryngology & Head and Neck Surgery, Saint-Luc University Hospital, Catholic University of Louvain, Avenue Hippocrate 10, 1200 Brussels, Belgium; 3 Department of Oto-Rhino-Laryngology & Head and Neck Surgery, CHU UCL Namur, Site de Godinne, Avenue Thérasse, 1, 5534 Yvoir, Belgium; philippe.eloy@chuuclnamur.uclouvain.be

**Keywords:** chronic rhinosinusitis, nasal polyps, vitamin D, narrative review

## Abstract

Chronic rhinosinusitis with nasal polyps (CRSwNP) is a subtype of chronic rhinosinusitis (CRS) characterized by bilateral nasal polyps, primarily affecting adults. It is often associated with hyposmia and asthma and driven by persistent Th2 inflammation, particularly in Caucasian patients. The disease is recurrent and significantly impacts quality of life, yet its pathophysiology remains poorly understood. Management includes intranasal steroids, short courses of systemic corticosteroids, surgery for refractory cases, and biologics. However, despite these treatment options, disease control remains challenging. Low vitamin D levels have been associated with worse clinical outcomes, while supplementation studies show promise in improving symptoms in deficient patients. Emerging research suggests that vitamin D modulates immunity, fibroblast activity, and epithelial integrity, potentially contributing to CRSwNP pathogenesis, though the exact mechanisms remain unclear. This review synthesizes current research on vitamin D’s role in systemic and local inflammation in CRSwNP. By highlighting its potential therapeutic implications, this work aims to guide future research and inform clinical practice. Additionally, it may serve as a foundation for understanding the broader impact of vitamin D deficiency in sinonasal diseases and other atopic conditions.

## 1. Introduction

Chronic rhinosinusitis (CRS) is defined as a chronic inflammation of the nose and paranasal sinuses, diagnosed when at least two symptoms are present—one of which must be either nasal blockage or nasal discharge, along with facial pain or pressure and/or a reduction or loss of smell. These clinical manifestations must persist for more than 12 weeks without resolution, and diagnosis requires confirmation through endoscopic findings or radiological signs on CT scan [1]. CRS is classified into two major subtypes: chronic rhinosinusitis with nasal polyps (CRSwNP), also known as nasal polyposis (NP); and chronic rhinosinusitis without nasal polyps (CRSsNP). These entities differ in clinical presentation and underlying cytokine profiles. Nasal polyposis is characterized by the presence of polyps in the nasal cavities and the middle meatus, observable during anterior rhinoscopy or nasal endoscopy. When based solely on symptoms or objective findings, CRS affects approximately 10–12% of the general population. However, using both criteria combined, the estimated prevalence drops below 5%, with approximately one-third of CRS patients having nasal polyps. Global prevalence shows considerable variability [2,3,4]. The exact prevalence of nasal polyposis (NP) is unclear, although estimates in Europe range from 2.1% to 4.3% [5]. NP is more frequently observed in Western countries, particularly in men over the age of 40, and in patients with asthma. It significantly reduces health-related quality of life and imposes a substantial socioeconomic burden.

In recent years, it has been recommended to categorize CRSwNP based on phenotypes and endotypes [6,7]. Phenotypes refer to the macroscopic appearance and anatomical distribution of nasal polyps. Endotypes, on the other hand, are defined by the underlying inflammatory mechanisms, including the nature of cellular infiltration and cytokine expression profiles. Histological analysis helps distinguish between eosinophilic and neutrophilic inflammation, which contributes to endotype classification. Neutrophilic inflammation predominates in East Asian patients and in conditions such as primary ciliary dyskinesia and cystic fibrosis—though these lie beyond the scope of this review—while eosinophilic polyps are more common in Caucasian populations. In Western countries, at least 80% of patients with CRSwNP exhibit a type 2 inflammatory cytokine profile, characterized by the presence of eosinophils and type 2 interleukins (IL)—such as IL-4, IL-5, IL-9, and IL-13—which are produced by Th2 (T-helper 2) lymphocytes and group 2 innate lymphoid cells (ILC-2) in the bloodstream or nasal mucosa [6,7,8]. Cytokines like IL-25, IL-31, IL-33, and thymic stromal lymphopoietin (TSLP), secreted by epithelial cells, are known to induce or amplify type 2 inflammation, resembling patterns seen in allergic rhinitis or asthma [9]. However, unlike allergic rhinitis, IgE levels in sensitized patients with CRSwNP are polyclonal. In contrast, patients with CRSwNP in East Asia or China often exhibit endotype 1 inflammation, characterized by elevated Th1 lymphocytes and IFN-γ, and/or endotype 3 inflammation involving Th17 lymphocytes, IL-17α, IL-6, and an increased number of neutrophils [10,11].

CRSwNP can be limited to the paranasal cavities and nasal fossae or be associated with systemic inflammatory diseases such as asthma, NSAID-exacerbated respiratory disease (Samter’s triad), vasculitis, allergic fungal sinusitis, and both allergic and non-allergic rhinitis. Asthma is equally frequent in non-allergic and allergic rhinitis but is more frequent in patients with polyposis. Aspirin sensitivity is also more common in nasal polyposis, independent of the allergic or non-allergic nature of rhinitis. Allergic rhinitis is frequently associated with nasal polyposis, mainly due to its high prevalence in the general population. However, “cellular” rhinitis forms such as NARES, NARMA, and NARESMA are much more frequently associated with nasal polyposis than true allergic rhinitis [12,13,14].

Despite a range of available therapies—including saline irrigations, topical corticosteroids, short courses of oral glucocorticoids, leukotriene antagonists, and macrolides—many patients require endoscopic sinus surgery (ESS) for adequate disease control. However, postoperative recurrence is frequent, with revision rates ranging from 20 to 50% [15]. Recent therapeutic breakthroughs have introduced biologic agents targeting the inflammatory pathways implicated in CRSwNP pathogenesis. Agents such as dupilumab (anti-IL-4/13), omalizumab (anti-IgE), mepolizumab and reslizumab (anti-IL-5), and benralizumab (anti-IL-5Rα) are now approved for use in selected populations. However, their widespread use remains limited by financial constraints, lack of reimbursement in some regions, and ongoing investigations into their long-term safety and efficacy [16,17].

CRSwNP is a multifactorial chronic inflammatory disease influenced by genetic predisposition, demographic factors, environmental exposures, and comorbidities. While anatomical variations, septal deviation, and humoral immunodeficiencies such as hypogammaglobulinemia are more commonly implicated in CRSsNP, factors such as obesity, gastroesophageal reflux disease (GERD), air pollution, and smoking are more frequently associated with CRSwNP and may contribute to both its development and persistence [18,19]. These triggers likely interact with individual host susceptibility to induce or perpetuate mucosal inflammation. Although the exact etiology of CRSwNP remains unclear, it is thought to involve a breakdown of the epithelial barrier in response to these environmental and genetic factors. Impaired production of antimicrobial peptides, dysregulated mucus secretion, and reduced epithelial integrity are among the key mechanisms contributing to sustained inflammation [19,20]. The involvement of microorganisms, particularly fungi and Staphylococcus aureus, in the pathogenesis of CRSwNP has been widely debated. The fungal hypothesis, initially proposed by Ponikau et al., suggests that common airborne fungi may elicit a strong eosinophilic immune response in predisposed individuals, leading to CRS [21,22,23,24]. This theory was extensively discussed in international forums; however, it remains controversial as subsequent studies failed to reproduce consistent results. Moreover, with improved fungal detection techniques, fungi have been found in both CRS patients and healthy controls [25,26,27].

More robust evidence supports the role of Staphylococcus aureus in CRSwNP [28,29,30,31,32,33,34,35,36]. This bacterium commonly colonizes the upper airways, with approximately 50% of the general population exhibiting intermittent nasal colonization. Notably, S. aureus is found in 64% of CRSwNP patients compared to 33% in non-polyp CRS patients and 20% in healthy individuals [31,37]. Certain strains of S. aureus produce enterotoxins that act as superantigens, triggering massive polyclonal T-cell activation and a strong Th2-type immune response. This can result in elevated IgE levels and persistent inflammation, especially in patients with severe asthma and frequent post-surgical recurrence.

Altogether, these mechanisms converge to sustain chronic sinonasal inflammation through persistent cytokine and chemokine expression. Among the various immunological factors involved, vitamin D has attracted growing interest. Controversy persists as to whether systemic vitamin D deficiency or sinonasal vitamin D alterations are a causal factor or a consequence of chronic inflammation, prompting ongoing debate in the field. This narrative review explores the potential immunomodulatory role of vitamin D in the pathogenesis and management of CRSwNP, examining its effects on innate and adaptive immune responses, epithelial function, and tissue remodeling. By synthesizing emerging evidence and identifying knowledge gaps, this review aims to determine whether optimizing vitamin D status could serve as a valuable adjunct to conventional treatments, contributing to a more comprehensive approach to CRSwNP management.

## 2. Vitamin D Physiology

Vitamin D is a secosteroid mainly formed from cholesterol (7-dehydrocholesterol) in cholecalciferol under the action of UVB (ultraviolet B). It can also be found in food, although in relatively small quantities, such as in oily fish, certain animal meats, or even certain mushrooms [38,39,40]. It exists in different forms depending on the level of hydroxylation, outlined below:

- Ergocalciférol (D2)/cholecalciferol (D3);
- Calcidiol, also called 25(OH)D3, after a first hydroxylation by a 25-hydroxylase. There are several enzymes capable of carrying out this chemical reaction but the one with the most affinity for vitamin D is CYP2R1, which is mainly found in the liver [41]. 25-hydroxylase (CYP2R1) is an unregulated enzyme, only dependent on its substrate: cholecalciferol (D3) or ergocalciferol (D2). Its activity could nevertheless decrease with age or obesity [42,43,44];
- Calcitriol, also called 1,25(OH)_2_D3, after a second renal hydroxylation with a 1-α-hydroxylase (CYP27B1) [45]. This enzyme is regulated by at least three hormones involved in phospho-calcium regulation: PTH, which stimulates it; and FGF-23 and 1,25(OH)D3, which inhibit it [43];
- The catabolic enzyme, 24-hydroxylase (CYP24A1), is able to transform 25(OH)D3 into 24,25(OH)_2_D3 and 1,25(OH)_2_D3 into 1,24,25(OH)_3_D3, two forms considered inactive.

Calcitriol (1,25(OH)_2_D3) is considered to be the active form of vitamin D. It has recently been proven that other tissues contain this 1-α-hydroxylase and, therefore, have the ability to locally convert vitamin D into its active form; this is particularly the case for epithelial cells of the respiratory mucosa or even immune cells (monocytes/macrophages, dendritic cells, T lymphocytes, and B lymphocytes) [46,47]. The kidney appears to be the primary, if not the only, source of circulating 1,25(OH)_2_D3 (i.e., in blood). An important characteristic of this extra-renal 1-α-hydroxylase is that its regulation differs from that of the kidney and that the extra-renal production of 1,25(OH)_2_D3 exerts autocrine and paracrine effects on the tissue from which it originates—this is regardless of 1,25(OH)_2_D3 blood levels or any other systemic endocrine control [48]. Vitamin D is traditionally known and used for its protective effect on the bone matrix. However, an increasing number of studies suggest that vitamin D has a widespread impact on the human body, with growing evidence of its role in the immune system (in infections, inflammation, neurodegenerative diseases, and autoimmune disorders), alongside its potential effects on cardiovascular health and carcinogenesis [49,50,51,52,53,54,55]. Indeed, active vitamin D is able to modulate the expression of certain genes by acting as a transcription factor at the nuclear level after having bound to its cellular vitamin D receptor (VDR). VDR is found in several cell types, particularly in nasosinus epithelial cells and in the immune system (including macrophages and antigen-presenting cells constitutionally, on T and B lymphocytes only after their activation, as well as on Treg cells).

## 3. Potential Role of Vitamin D in the Pathogenesis of CRSwNP

**Figure 1:**
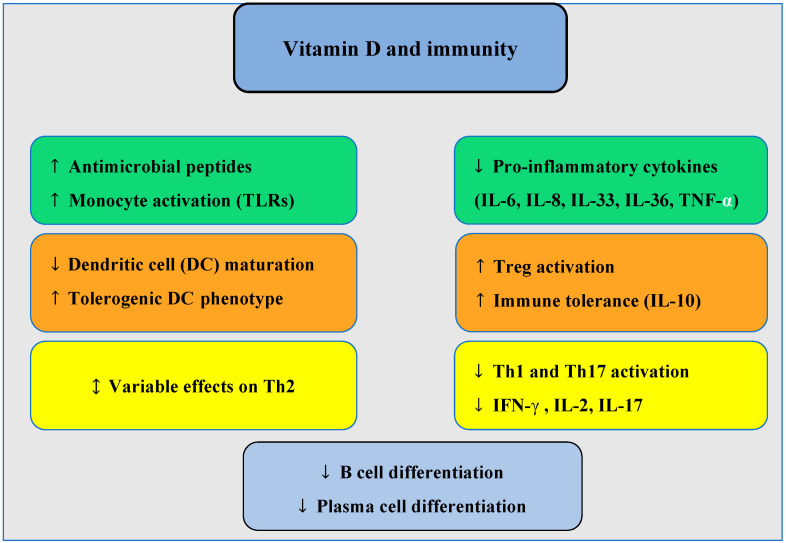
Schematic summary of the main effects of vitamin D on innate and adaptive immunity, as described in Section 3. ↑ indicates increase or activation; ↓ indicates a decrease or inhibition; ↕ indicates variable or context-dependent effects.

It is widely recognized that vitamin D plays a key role in stimulating innate immunity and modulating acquired immunity (see Figure 1). Specifically, in the context of innate immunity, monocytes increase their 1-α-hydroxylase activity, and the vitamin D receptor (VDR) is upregulated following stimulation of Toll-like receptors (TLRs). 1,25(OH)_2_D3 enhances their production of antimicrobial peptides, such as cathelicidin and defensin Beta [56], while also increasing monocyte chemotaxis and phagocytosis [57]. Additionally, 1,25(OH)_2_D3 has been shown to reduce the production of pro-inflammatory cytokines by type 2 innate lymphoid cells (ILC2) [58]. At the dendritic cell level (in both innate and adaptive immunity), vitamin D plays a crucial role in antigen maturation and presentation during TLR stimulation. It also supports the production of cathelicidin—vital for innate antibacterial responses [59]. In terms of acquired immunity, 1,25(OH)_2_D3 exhibits a tolerogenic effect by promoting the development of plasmacytoid dendritic cells and reducing the number of myeloid dendritic cells, which are responsible for activating lymphocytes [60]. Interestingly, naive T and B lymphocytes do not initially express VDR but its expression increases when these cells are stimulated by mitogens. 1,25(OH)_2_D3 directly inhibits lymphocyte proliferation and indirectly by modulating dendritic cells [60]. The direct effects of vitamin D on Th2 cells remain somewhat inconsistent, with both stimulatory and inhibitory effects on Th2 cell cytokines reported in human and animal studies. Current research suggests that elevated 25(OH)D3 levels do not increase blood Th2 lymphocytes [61]. 1,25(OH)_2_D3 is known to reduce Th1 lymphocyte development, increase IL-10 (a recognized modulator of the immune response), and is thought to enhance IL-4 production. It decreases the levels of IFN-γ, IL-2, IL-6, IL-33, IL-36, and TNF-α, while reducing LTh17 production and promoting the formation of regulatory T lymphocytes (Treg) [47,62,63]. At the level of B lymphocytes, vitamin D decreases their proliferation and inhibits plasma cell differentiation [64,65].

Beyond its immune effects, vitamin D positively influences epithelial barrier function and the respiratory tract microbiota [47,66]. Active vitamin D regulates over 2000 genes in nasal epithelial cells [63]. 1,25(OH)_2_D3 appears to affect fibroblasts, which are critical in the development of nasal polyps [67]. Exposure of nasal polyp fibroblasts to 1,25(OH)_2_D_3_ reduces the IL-1β-induced release of eosinophil-attracting chemokines (RANTES and eotaxin), decreases the TNF-α-induced expression of matrix metalloproteinases 2 and 9 (MMP2 and MMP9)—which play a significant role in airway tissue remodeling—and also reduces IL-6 and IL-8 induced by lipopolysaccharides [68,69,70]. Vitamin D also inhibits fibrotic changes associated with myofibroblast differentiation and excessive extracellular matrix production in nasal-polyp-derived fibroblasts stimulated by TGF-β1 [71]. Moreover, the anti-proliferative effect of topical corticosteroids on nasal polyp fibroblasts appears to be potentiated by the addition of 1,25(OH)_2_D_3_ [72,73,74]. In asthmatic patients, several studies indicate that combining corticosteroids with vitamin D is particularly beneficial in patients with vitamin D deficiency. In such cases, larger doses of glucocorticoids may be required to achieve therapeutic effects [75,76].

## 4. Sinonasal Vitamin D Metabolism in CRSwNP

Although limited in number and employing varied analytical techniques, current biochemical studies on enzyme expression in the sinonasal tissues of patients with chronic rhinosinusitis with nasal polyps (CRSwNP) provide valuable insights into the possible role of vitamin D metabolism in the pathophysiology of this disease (see Table 1). Among the five studies analyzed, four reported a reduced expression of CYP27B1 (1-α-hydroxylase), the enzyme responsible for converting inactive vitamin D (25(OH)D_3_) into its active form (1,25(OH)_2_D_3_). This reduction has been observed both at the RNA and protein levels, highlighting a local defect in vitamin D activation in CRSwNP tissues. This enzymatic deficiency is considered a key factor contributing to the reduced local availability of active vitamin D, which could exacerbate sinonasal inflammation.

The expression of local CYP24A1, the enzyme that inactivates vitamin D, shows variability across studies. One study reported a significant increase in RNA expression, suggesting an accelerated turnover of active vitamin D in CRSwNP tissues, while another study observed a decrease [63,77]. However, CYP24A1 was not studied in the other investigations, leaving its role in CRSwNP insufficiently characterized. These conflicting findings underscore the need for further research -on the regulation and function of this enzyme in sinonasal tissues.

The expression of the vitamin D receptor (VDR), which mediates the biological effects of active vitamin D, might also be altered in CRSwNP. Some studies have reported a significant reduction in both RNA and protein levels of VDR in sinonasal tissues, while others have found a decrease at the protein level but no difference at the RNA level [63,78]. Additionally, several investigations have reported no significant changes in VDR gene expression [77,79]. These discrepancies likely reflect differences in cohort characteristics, analytical methods, or sample sizes. Interestingly, the observed reduction in VDR protein levels, despite unchanged RNA expression in some studies, could suggest the involvement of post-transcriptional or post-translational regulatory mechanisms. Nevertheless, when VDR expression is reduced, it may indicate a diminished capacity of sinonasal tissues to respond to active vitamin D, further compounding the functional impact of reduced CYP27B1 activity.

Beyond enzymatic regulation, both systemic and local vitamin D levels have been investigated to better understand their role in CRSwNP pathophysiology. Findings on serum 25(OH)D_3_ levels remain inconsistent, with some studies reporting significantly lower levels in CRSwNP patients compared to controls, while others found no significant difference. However, a positive correlation has been observed between serum 25(OH)D_3_ levels and sinonasal VDR gene expression [77]. At the local level, data on sinonasal 25(OH)D_3_ are more limited but suggest a clear trend toward reduced levels in CRSwNP tissues [63,79]. While systemic serum levels of the active form, 1,25(OH)_2_D_3_, appear to be generally preserved, sinonasal levels show a marked decrease [79,80]. This local reduction likely reflects impaired CYP27B1 activity, leading to insufficient conversion of 25(OH)D_3_ into its active form at the tissue level.

These findings suggest that CRSwNP may be associated with dysregulated vitamin D metabolism, primarily involving impaired CYP27B1 (1-α-hydroxylase) activity, leading to reduced levels of its active form, 1,25(OH)_2_D_3_. The role of CYP24A1 in vitamin D inactivation remains uncertain due to limited and contradictory evidence. Similarly, alterations in vitamin D receptor (VDR) expression, particularly the observed reduction at the protein level despite stable RNA expression, suggest a potential impairment in vitamin D signaling that warrants further investigation. Local deficiencies in sinonasal vitamin D, combined with inconsistent systemic alterations, may contribute to disease pathophysiology by limiting the effects of active vitamin D. However, the precise interplay between systemic and local vitamin D metabolism—as well as the regulation of CYP24A1 and VDR—remains poorly understood, highlighting the need for further research.

**Table 1:** Sinonasal vitamin D metabolism in CRSwNP patients.

	Christensen et al. 2017 [77]	Schlosser et al. 2016 [80]	Tomawska et al. 2018 [78]	Xiao et al. 2024 [63]	Mulligan et al. 2014 [79]
**Number of patients**	32 total (10 CRSwNP, 8 CRSsNP, 13 controls)	50 total (13 CRSwNP, 13 CRSsNP, 18 controls)	166 total (55 CRSwNP, 52 CRSsNP, 59 controls)	212 total (70 eosinophilic CRSwNP, 67 non-eosinophilic CRSwNP, 75 controls); sub-cohorts: - 54 CRSwNP + 12 controls for PCR - 18 CRSwNP + 9 controls for Western blot - 67 CRSwNP + 18 controls for immunohistochemistry	37 total (14 CRSwNP, 12 CRSsNP, 11 controls); sub-cohorts: - 6 CRSwNP + 5 controls (smoke-naive) for RNA (RT-PCR) - 4 CRSwNP + 4 controls (smoke-naive) for immunohistochemistry
**1-α-hydroxylase (CYP27B1)**	Reduction in gene expression (RNA) but not statistically significant	Significant decrease in protein expression	No significant difference in protein and gene expression	Significant decrease in both RNA and protein levels	Significant decrease in RNA expression
**Vitamin D receptor (VDR)**	No difference in gene expression (RNA)	Not studied	Significantly lower protein levels; no change in gene expression	Significant decrease in both protein and RNA levels	No difference in gene expression
**25-hydroxylase (CYP2R1)**	No difference in gene expression (RNA)	Not studied	Not studied	Not studied	No difference in gene expression (RNA)
**24-hydroxylase (CYP24A1)**	Significant increase in RNA expression	Not studied	Not studied	Significant decrease in RNA expression	Not studied
**Vitamin D levels**	Positive significant correlation between serum 25(OH)D and VDR expression but not with CYP2R1, CYP27B1, and CYP24A1	Serum 25(OH)D3 and sinonasal 1,25(OH)_2_D3 levels were significantly lower. No serum 1,25(OH)_2_D3 differences	No 25(OH)D3 serum difference	Serum and sinonasal tissue 25(OH)D3 levels were significantly lower	Serum 25(OH)D3, sinonasal tissue 25(OH)D3, and 1,25(OH)_2_D3 levels were significantly lower
**Technique used**	RT-PCR	Immunohistochemistry + flow cytometry	Immunohistochemistry with H-score quantification + DNA microarray technique	RT-PCR, Western Blot, immunohistochemistry, ELISA	RT-PCR, ELISA, immunohistochemistry + flow cytometry

## 5. Vitamin D and Clinical CRSwNP Studies

Vitamin D has been extensively studied in the context of chronic rhinosinusitis with nasal polyps (CRSwNP), with growing evidence supporting its role in disease pathophysiology and management. Experimental models indicate that vitamin D deficiency induces immune alterations in the sinonasal mucosa, resembling those observed in atopic airway inflammation. Additionally, inflammation associated with Aspergillus-fumigatus-induced chronic rhinosinusitis (Af-CRS) suppresses sinonasal vitamin D metabolism, leading to a reduction in local levels of its active metabolite, 1,25(OH)_2_D_3_. Dietary vitamin D deficiency further exacerbates these immunological alterations [81]. At the clinical level, lower serum 1,25(OH)_2_D_3_ levels have been correlated with increased sinonasal fibroblast proliferation, as indicated by markers such as vimentin and Ki67, suggesting a potential role of vitamin D in tissue remodeling and inflammation [82].

Retrospective studies have demonstrated a negative correlation between serum 25(OH)D_3_ levels in CRSwNP patients and both the Lund–Mackay score and the SNOT-22 score, reinforcing the link between vitamin D deficiency and disease severity [83,84]. Additionally, one of these studies suggested that 25(OH)D_3_ serum levels may influence symptom improvement after surgery [84].

Prospective case-control, observational, and cross-sectional studies have shown that CRSwNP patients have significantly lower 25(OH)D_3_ levels compared to controls. These studies also report a significant association between 25(OH)D_3_ levels and nasal polyp grade, as well as a negative correlation with CT findings and subjective nasal severity (SNOT-22), further supporting the recommendation of measuring vitamin D levels as part of the initial assessment for these patients [85,86,87]. Other cross-sectional studies have reached similar conclusions [88,89]. Additional evidence suggests that vitamin D deficiency is also associated with systemic inflammation, as demonstrated by a statistically significant negative correlation between hs-CRP levels and serum 25(OH)D_3_. These findings indicate that lower vitamin D levels are linked to increased inflammatory activity in CRSwNP patients [89]. Given these findings, some researchers advocate for routine vitamin D screening as part of CRSwNP management [87,88]. Recent systematic reviews and meta-analyses reinforce these findings by demonstrating that CRSwNP patients have significantly lower serum 25(OH)D_3_ levels compared to controls. They also highlighted that low vitamin D levels are correlated with greater disease severity and intense clinical manifestations [90,91,92].

Beyond these observational associations, interventional studies suggest that vitamin D supplementation may have therapeutic benefits. A triple-blind interventional study showed that postoperative supplementation with 4000 IU (International Unit)/day of vitamin D (cholecalciferol) to conventional treatment reduced polyp recurrence rates and improved symptom scores, with a reported 65.75% reduction in SNOT-22 scores and an 86.15% decrease in Meltzer scores at six months post-surgery in patients with pre-existing vitamin D insufficiency compared to controls [93]. Another trial suggests that weekly supplementation of 60,000 IU vitamin D over three months leads to symptom improvement in vitamin-D-deficient patients, though interpretation is limited due to study heterogeneity, particularly regarding the inclusion of patients without nasal polyps [94]. Emerging evidence suggests that endoscopic sinus surgery itself may influence vitamin D metabolism, with postoperative increases in serum 25(OH)D_3_ levels observed in some studies. This raises the possibility that reducing sinonasal inflammation could help restore systemic vitamin D homeostasis [95]. In addition, another study demonstrated that patients with isolated nasal polyposis or nasal polyposis associated with cystic fibrosis had lower serum vitamin D levels compared to those with chronic sinusitis without polyps or cystic fibrosis without nasal polyposis. They found a significant inverse correlation between these serum levels, the Lund–Mackay score, and sinonasal bacterial colonization, highlighting the potential role of vitamin D in innate immunity and epithelial barrier function [96].

Randomized controlled trials evaluating high-dose vitamin D supplementation have demonstrated improvements in both clinical and radiological outcomes. A double-blind randomized trial in CRSwNP patients with vitamin D deficiency showed that monthly intramuscular administration of 200,000 IU of cholecalciferol until normalization significantly improved Meltzer grading scores and CT scan findings [97]. Similarly, a recent triple-blinded, placebo-controlled study in vitamin-D-deficient CRSwNP patients demonstrated significant postoperative improvements in both objective (Meltzer score) and subjective (SNOT-22) outcomes in the supplemented group compared to controls [98]. Additionally, a non-randomized study found that administering 50,000 IU of vitamin D weekly for 8 weeks post-surgery in CRSwNP patients with 25(OH)D3 insufficiency or deficiency reduced polyp recurrence by nearly 50% at 6 months, along with a significantly greater reduction in SNOT-22 scores [99]. Even short-term supplementation with 4000 IU/day of vitamin D for one month has been reported to reduce CRSwNP symptoms and polyp size significantly [100].

**Figure 2:**
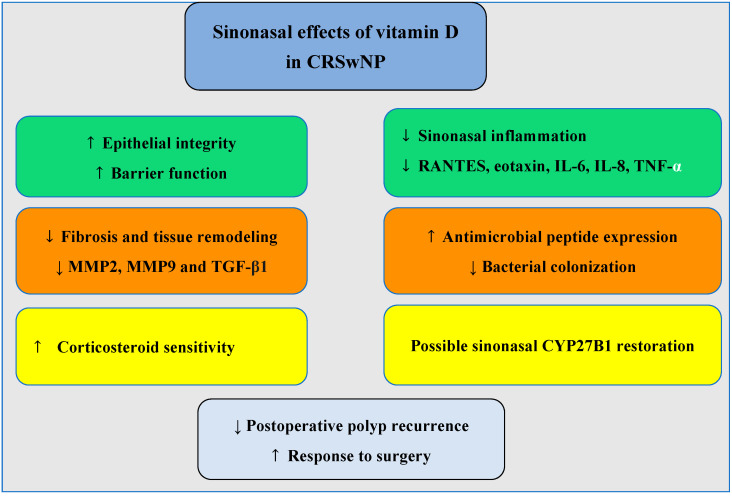
↑ indicates increase or activation; ↓ indicates a decrease or inhibition. Schematic representation of the possible immunological, epithelial, and clinical effects of vitamin D in chronic rhinosinusitis with nasal polyps (CRSwNP). These include reduced sinonasal inflammation (RANTES, eotaxin, IL-6, IL-8, and TNF-α); inhibition of tissue remodeling and fibrosis (downregulation of MMP2, MMP9, and TGF-β1); improved epithelial barrier function; enhanced antimicrobial defense; increased corticosteroid sensitivity; and reduced postoperative recurrence with 25(OH)D_3_ supplementation. Potential local CYP27B1 activity restoration may also occur through inflammation control.

These findings collectively suggest that vitamin D deficiency may contribute to the pathophysiology of CRSwNP and that supplementation, particularly in deficient patients, could have a role in disease management (see Figure 2 for a visual summary of the sinonasal effects of vitamin D in CRSwNP). Further research, including well-designed randomized controlled trials, is needed to establish optimal dosing strategies and clarify the long-term impact of vitamin D on disease progression and recurrence.

## 6. Serum Vitamin D Levels

The ideal blood levels and daily amount of vitamin D needed to benefit from its pleiotropic effects are still debated. Disease-specific practice guidelines are currently unavailable. For instance, the 2024 Endocrine Society Clinical Practice Guideline for 25(OH)D3 treatment and serum testing recommended against routine 25(OH)D screening in healthy adults, citing insufficient evidence for specific thresholds to guide treatment and uncertain benefits. Concerns included feasibility, cost, and the lack of a universal threshold for all populations [101]. A 2025 review conducted by four leading vitamin D experts already criticized these new recommendations and opposed them. They argued that these guidelines are bone-centric, largely overlooking the extra-skeletal benefits of vitamin D, and that they rely exclusively on specific types of studies, disregarding broader evidence. They also highlighted that 25% of Americans and 60% of Central Europeans have deficient vitamin D levels (<20 ng/mL), reinforcing the need for a more comprehensive approach [102]. This ongoing debate underscores the persistent controversy within the scientific community. However, the current Central European guidelines were developed in recognition of the evidence supporting both the skeletal and pleiotropic effects of vitamin D, making them relevant to clinical practice. These guidelines recommend vitamin D supplementation to achieve and maintain a target 25(OH)D3 concentration between 30 and 50 ng/mL (75 to 125 nmol/L) [103]. The American Endocrine Society recommends maintaining serum 25(OH)D3 concentrations greater than 30 ng/mL (>75 nmol/L), with a preferred range of 40 to 60 ng/mL (100 to 150 nmol/L) [21]. The 2025 expert consensus and review also suggest maintaining serum concentrations above 40 ng/mL to fully benefit from the extra-skeletal effects of vitamin D, with an upper threshold of 70 ng/mL [102]. It is generally accepted that a serum concentration of up to 100 ng/mL (250 nmol/L) is safe for both children and adults, although there is currently no evidence that such levels bring more benefits [104]. According to the Endocrine Society’s guidelines, vitamin D toxicity is extremely rare, with a 25(OH)D3 concentration of at least 150 ng/mL (375 nmol/L) required to show evidence of toxicity [105].

To maintain sufficient levels in adults, supplementation of 800 to 2000 IU/day (20.0 to 50.0 μg/day) is recommended between October and April, depending on body weight. Year-round supplementation is advised if sufficient skin synthesis of vitamin D cannot be ensured during the summer. For the elderly and adults with obesity (BMI > 30), supplementation of 1000 to 4000 IU/day (25 to 100 μg/day) is recommended throughout the year [103,106].

For adults and the elderly with deficient vitamin D levels (<20 ng/mL), supplementation of 7000 to 10,000 IU/day (175 to 250 μg/day) or 50,000 IU/week (1250 μg/week) is recommended as the maximum allowable dose. It is reasonable to reassess the 25(OH)D3 concentration after 3 to 4 months and monitor it semi-annually, particularly in patients with factors like obesity that may require higher therapeutic doses. To maintain a target level of 40–60 ng/mL, the equivalent of 4000 to 5000 IU daily is required [103].

Special attention should be given to patients with granulomatous diseases (e.g., sarcoidosis, Wegener’s granulomatosis, and chronic fungal infections) and certain lymphomas as they are at risk for developing hypercalcemia. For these patients, the target level should remain between 20 and 30 ng/mL. Patients with severe renal and hepatic impairment also require special attention as they may need “active” forms of vitamin D such as 1,25(OH)_2_D3 or 25(OH)D3, respectively.

## 7. Discussion

Epidemiological evidence has increasingly highlighted the relevance of vitamin D in chronic rhinosinusitis with nasal polyps (CRSwNP). Several recent systematic reviews and meta-analyses have shown that patients with CRSwNP generally exhibit lower serum 25(OH)D_3_ levels compared to healthy controls, with deficiency occurring more frequently and being more strongly correlated with disease severity. This has drawn attention to its immunomodulatory effects on adaptive immunity, its activation of innate immunity, its regulation of fibroblasts, and its potential roles in strengthening epithelial barriers and modulating the upper airway microbiota. Furthermore, vitamin D deficiency has been associated with atopic conditions such as asthma and allergic rhinitis, both of which frequently coexist with CRSwNP and share similar cellular profiles with the predominant Western CRSwNP endotype (eosinophilic, type 2 inflammation), which may represent a modifiable factor contributing to disease management in vitamin-D-deficient patients [107,108,109,110,111,112,113].

An inverse relationship between serum 25(OH)D_3_ levels and both symptom severity and mucosal involvement in CRSwNP patients has been observed. While prospective interventional studies remain limited, initial findings are encouraging. Nevertheless, it remains unclear whether systemic inflammation stems from 25(OH)D_3_ insufficiency or if inflammation lowers 25(OH)D_3_ levels due to its metabolic conversion into 1,25(OH)_2_D_3_ to modulate inflammation [114,115]. One study has suggested that surgical interventions reducing inflammation can increase serum 25(OH)D_3_ levels. This finding underscores the potential bidirectional relationship between systemic vitamin D levels and inflammation. Furthermore, interventional postoperative studies have highlighted the benefits of adding oral vitamin D supplementation after surgery to prevent recurrence and maintain anti-inflammatory effects. These observations emphasize the importance of investigating whether systemic supplementation could indirectly influence sinonasal vitamin D metabolism and modulate local inflammation. As with the possible systemic relationship, local interactions between vitamin D metabolism and sinonasal inflammation also appear to be bidirectional. On one hand, inflammatory processes may impair the production of active vitamin D (1,25(OH)_2_D_3_) in sinonasal tissues, while on the other hand, a deficiency in active vitamin D may perpetuate local inflammation. These disruptions, which include impaired activity of 1-α-hydroxylase and reduced binding efficiency of the vitamin D receptor (VDR), underscore the complexity of local vitamin D pathways in CRSwNP. Moreover, conflicting findings regarding CYP24A1, the enzyme responsible for inactivating vitamin D, further highlight the need to clarify the regulatory mechanisms underlying local metabolic disturbances.

The accessibility of the nasal cavity provides unique opportunities for localized treatments. Topical applications of calcitriol or its analogs, which have shown efficacy in reducing inflammation in other conditions such as psoriasis, could directly target sinonasal inflammation [116,117]. Studies have shown that exposing nasal polyp fibroblasts to 1,25(OH)_2_D_3_ reduces the release of eosinophil-attracting chemokines, decreases fibroblast proliferation, and may influence airway remodeling. These effects appear to be synergistic when combined with corticosteroids, potentially extending to the regulation of other pro-inflammatory cytokines released from nasal polyps [63,68,69,70,71,72,73,74]. Taking into account the potential systemic absorption through the nasal mucosa, integrating vitamin D analogs with standard anti-inflammatory therapies could therefore offer an improved strategy for managing CRSwNP, while ensuring careful monitoring of possible systemic effects [118].

Finally, determining whether a combination of systemic and localized therapies is more effective than either approach alone remains a critical question. Systemic supplementation aims to correct serum 25(OH)D_3_ insufficiency, which may indirectly reduce systemic inflammation and provide a substrate for any remaining local enzymatic activity. On the other hand, localized delivery of vitamin D analogs, such as calcitriol, could bypass the need for local enzymatic conversion and directly target sinonasal inflammation. However, topical treatments have anatomical limitations in their ability to fully penetrate inflamed or remodeled sinonasal tissues, potentially leaving certain areas untreated. In such cases, normalizing systemic vitamin D levels could provide additional support by addressing regions inaccessible to topical applications and maintaining a broader anti-inflammatory effect throughout the body. This complementary relationship is particularly important given the complexity of vitamin D metabolism in CRSwNP. While the enzymatic activity of CYP27B1 may be impaired in inflamed sinonasal tissues, systemic supplementation ensures an adequate supply of 25(OH)D_3_, which may still be metabolized by areas with residual enzymatic function. Moreover, systemic supplementation could alleviate systemic inflammation, which might otherwise perpetuate local dysfunctions, creating a potential bidirectional benefit.

Another emerging area of interest involves genetic variations in vitamin D metabolism, specifically polymorphisms in the vitamin D receptor (VDR), which could significantly influence therapeutic responses [119]. These genetic differences may affect both the binding affinity of vitamin D to its receptor and the downstream transcriptional activity that mediates its anti-inflammatory and immunomodulatory effects. Exploring such polymorphisms could not only clarify interindividual variability in disease progression and treatment outcomes but could also provide a basis for developing personalized treatment strategies. Future research into the prevalence and impact of these genetic factors in CRSwNP populations could pave the way for targeted therapies that optimize vitamin-D-related interventions. Future studies should focus on whether higher systemic vitamin D levels, localized delivery of vitamin D analogs, or combined strategies can effectively modulate sinonasal metabolic dysfunction, reduce inflammation, and prevent recurrence. Understanding the complex interplay between systemic and local vitamin D pathways, as well as incorporating genetic insights into treatment plans, is essential for advancing personalized treatment strategies for CRSwNP patients.

It is also important to consider the broader clinical applicability and potential economic value of vitamin D supplementation as a simple, accessible adjunctive intervention. From an economic perspective, vitamin D supplementation may represent a cost-effective adjunctive strategy in the management of CRSwNP, particularly in patients with deficiency. Its low cost, favorable safety profile, and growing clinical evidence—suggesting reduced symptom burden and potentially lower rates of postoperative recurrence—indicate that it could help reduce the need for repeated surgeries and prolonged systemic treatments. Moreover, maintaining adequate serum 25(OH)D_3_ levels may offer broader public health benefits, including improved bone health, enhanced immune defense against infections, and modulation of chronic inflammation. Although specific cost-effectiveness analyses in CRSwNP are currently lacking, these considerations support further research into the clinical and economic relevance of this approach in this patient population.

Although this review has primarily focused on CRSwNP, it is important to briefly address the related but distinct entity of CRSsNP. Several recent systematic reviews and meta-analyses also indicate vitamin D deficiency in chronic rhinosinusitis without nasal polyps (CRSsNP), though this deficiency appears consistently more pronounced and more strongly correlated with clinical severity in CRSwNP [90,91,92]. Additionally, studies specifically investigating local vitamin D metabolism suggest more significant alterations in enzymatic activity (CYP27B1, CYP24A1) and abnormalities in vitamin D receptor (VDR) expression in CRSwNP tissues compared to CRSsNP [63,77,78,79,80]. Nevertheless, the CRSsNP phenotype remains comparatively less explored in the current literature, justifying our specific focus on CRSwNP and highlighting the need for further comparative studies to better characterize these phenotypic differences.

## 8. Conclusions

Endotyping has become essential for understanding the diverse inflammatory mechanisms in nasal polyposis and for tailoring treatment strategies to individual patients. Incorporating the optimization of serum vitamin D levels into a holistic management approach for CRSwNP appears promising and may enhance patient outcomes. Maintaining serum 25(OH)D_3_ concentrations within the 40–60 ng/mL range could serve as an adjunct to conventional therapies, with potential systemic and local anti-inflammatory benefits. However, the precise interaction between systemic vitamin D levels and sinonasal inflammation remains unclear, emphasizing the need for further research into this bidirectional relationship. Future investigations should aim to clarify the local versus systemic effects of vitamin D, evaluate the efficacy of targeted topical applications of calcitriol or its derivatives, and explore personalized treatment approaches informed by genetic profiling, such as VDR polymorphisms. These studies could yield innovative strategies to better manage CRSwNP, reduce inflammation, and prevent recurrence, ultimately paving the way for more effective, patient-centered care.

## Abbreviations

The following abbreviations are used in this manuscript:

CRSwNP	Chronic rhinosinusitis with nasal polyps
CRSsNP	Chronic rhinosinusitis without nasal polyps
IL	Interleukin
NSAID	Non-steroidal anti-inflammatory drug
Th	T helper
NARES	Non-allergic rhinitis with eosinophils
NARMA	Non-allergic rhinitis with mast cells
NARESMA	Non-allergic rhinitis with eosinophils and mast cells
IFN	Interferon gamma
CYP	Cytochrome P450
TNF-α	Tumor Necrosis Factor-alpha
TGF-β1	Transforming Growth Factor-beta 1
PTH	Parathyroid hormone
FGF	Fibroblast Growth Factor
TLR	Toll-like receptors
VDR	Vitamin D receptor
PCR	Polymerase Chain Reaction
RNA	Ribonucleic Acid
IU	International Unit
CT scan	Computed tomography scan
SNOT-22	Sinonasal Outcome Test-22
BMI	Body Mass Index
